# Genome-Wide Gene Expression Profiling Reveals the Direct Effect of Dienogest on Ovarian Endometriotic Stromal Cells

**DOI:** 10.1007/s43032-023-01181-4

**Published:** 2023-02-08

**Authors:** Hiroshi Honda, Norihisa Nishimichi, Mayumi Kaneko, Michinori Yamashita, Yumiko Akimoto, Hirotoshi Tanimoto, Mitsue Teramoto, Hideki Teramoto, Yasuyuki Yokosaki

**Affiliations:** 1 Department of Obstetrics and Gynecology, Hiroshima City North Medical Center Asa Citizens Hospital, 1-2-1 Kameyamaminami, Asakita-ku, Hiroshima, 731-0293 Japan; 2 Integrin-Matrix Biomedical Science, Translational Research Center, Hiroshima University, Hiroshima, Japan; 3 Department of Diagnostic Pathology, Hiroshima City North Medical Center Asa Citizens Hospital, Hiroshima, Japan; 4 Department of Surgery and Palliative Medicine, Fujita Health University School of Medicine, Toyoake, Japan; 5 Sumire Women’s Clinic, Hiroshima, Japan; 6 Department of Obstetrics and Gynecology, Sera Central Hospital, Sera, Hiroshima, Japan; 7 Department of Obstetrics and Gynecology, Shobara Red Cross Hospital, Shobara, Japan

**Keywords:** Endometriosis, Dienogest, Microarray, Gene ontology, Ingenuity pathway analysis, MMP

## Abstract

Endometriosis affects up to 10% of women of reproductive age, causing dysmenorrhea, chronic pelvic pain, and infertility. The current key drug for endometriosis is dienogest, a progestin with high specificity for the progesterone receptor. To reveal the direct anti-endometriotic effect of dienogest on ovarian endometriotic cells, we investigated the genome-wide gene expression profiles of ovarian endometriotic stromal cells with (Dienogest group) or without dienogest treatment (Control group) and compared the groups’ gene expression profiles. We performed a gene ontology (GO) analysis and Ingenuity pathway analysis using these data. To validate the microarray data, we performed real-time RT-PCRs and immunohistochemistry for the differentially expressed genes between the two groups. Of 647 genes differentially expressed between the two groups, 314 genes were upregulated and 333 were downregulated in the Dienogest group versus the Control group. The GO analysis showed that the regulation of macrophage chemotaxis, the collagen catabolic process, and the proteoglycan biosynthetic process are the main biological processes closely associated with the differentially expressed genes. We identified 20 canonical pathways that were most significantly differentially expressed in the Dienogest group versus the Control group. We observed that matrix metalloproteinases (MMPs) are the genes in these pathways that are most closely associated with dienogest treatment. Of components involved in the regulation of macrophage chemotaxis, colony-stimulating factor 1 and macrophage-stimulating 1 are potential upstream regulators of MMPs and were observed herein to be suppressed by dienogest. Our results suggest that dienogest may thus exert its anti-endometriotic effect by directly suppressing MMPs.

## Introduction

Endometriosis, which is defined by the presence of ectopic endometrial tissue outside the uterus, is a chronic disease affecting up to 10% of women of reproductive age [1]. Infertility and chronic pelvic pain are the main symptoms of endometriosis. For chronic pelvic pain, mainly hormonal therapies have been provided in the past decades, particularly therapies involving gonadotropin-releasing hormone (GnRH) analogs. However, GnRH analogs cannot be used for a long period because of their side effects, such as bone loss. It has thus been difficult for individuals with chronic pelvic pain due to endometriosis to obtain relief from pain over a long period by using a GnRH analog.

Dienogest is classified as a fourth-generation progestin with high specificity for the progesterone receptor. It has an effect on endometriosis-induced pain that is equivalent to that of a GnRH analog [2]. However, unlike GnRH analogs, dienogest can be used for long periods and has brought endometriosis patients long-term relief from pain [3]. Dienogest has thus replaced GnRH analogs as the key drug of hormonal therapies for endometriosis. The anti-endometriotic effect of dienogest is thought to be due mainly to its ability to suppress ovulation [4, 5], and several studies have shown that dienogest directly inhibits the inflammatory responses or aromatase expression in endometriotic cells [6–8]. Another investigation indicated that dienogest directly inhibits the proliferation of ovarian endometriotic stromal cells (ESCs) [9]. Although many studies have attempted to reveal the biological mechanisms that underlie the effects of dienogest on endometriosis, the precise mechanisms remain unknown.

Genome-wide gene expression profiling, using a microarray, and its subsequent pathway analysis have revealed novel biological cell-related findings for several diseases. However, to the best of our knowledge, no reports of genome-wide gene expression profiling to investigate the biological actions of dienogest in endometriotic cells have been published.

We conducted the present study to determine the genome-wide gene expression profile of ovarian ESCs treated with dienogest. We also performed a pathway analysis using the data of the gene expression profile. The results demonstrated that, in ovarian ESCs, matrix metalloproteinases (MMPs) were particularly important among the genes that are directly modified by dienogest.

## Materials and Methods

### Patients and Samples

Tissue specimens of ovarian endometriotic cysts were obtained during gynecological surgeries from 15 patients (5 for the present microarray analysis and 10 for immunohistochemistry) with stage III–IV endometriosis evaluated in accord with the American Society for Reproductive Medicine classification of endometriosis. Before the surgeries, the patients provided written informed consent for their materials to be used. All 15 patients were of reproductive age (age range 24–46 years old, mean ± SD: 34.9 ± 8.5 years old) with a body mass index in the normal range (mean ± SD: 21.3 ± 2.6), had regular menstrual cycles, and were clinically and pathologically confirmed to have no gynecological disease other than endometriosis. None of the five patients for the microarray analysis had received any hormonal therapies and, of the 10 patients whose specimens were used for immunohistochemistry, five had also not received any hormonal therapies; the other five patients had received dienogest in oral doses (1 mg twice a day) for 12–18 weeks (mean ± SD: 15 ± 2.2 weeks) months until the surgery. All specimens were pathologically confirmed as ovarian endometriotic cyst tissues after the surgeries.

The protocol of the present study was approved by the Ethical Committee of Hiroshima City Asa Citizens Hospital, and all experiments were carried out in accord with relevant guidelines and regulations.

### Isolation and Cell Culture of Ovarian ESCs

The isolation of ovarian ESCs was performed as described by Honda et al. [10]. Briefly, endometriotic tissue layers were scraped from the inner wall of the cyst, minced into small pieces, and enzymatically dissociated by incubation with 0.25% collagenase (Sigma-Aldrich, St. Louis, MO, USA) and 0.02% DNase I (Sigma-Aldrich) in phenol-red-free Dulbecco’s modified Eagle’s Medium/Ham’s F-12 (Invitrogen, Carlsbad, CA) supplemented with 10% charcoal-stripped fetal bovine serum (FBS) (Invitrogen) for 1 h at 37 °C in an atmosphere of 5% CO_2_ under magnetically driven agitation. Enrichment of the ESCs was performed by serial filtration using 100 μm and 40 μm nylon sieves (BD Falcon, Franklin Lakes, NJ), and filtered cells were collected onto two 6 cm culture dishes per sample. After incubation of the filtered cells at 37 °C for 30 min to allow the ESCs to attach to the dishes, the media were removed, and the dishes were washed for complete removal of the floating endometriotic epithelial cells and other cells such as blood cells in the supernatant.

The ESCs were cultured in DMEM/Ham’s F-12 medium supplemented with 10% charcoal-stripped FBS and 1% penicillin and streptomycin (100 mg/ml) (Invitrogen) under the conditions described above. The medium was changed every other day and, when the cells reached 80% confluence, the culture was serum-starved in serum-free DMEM/Ham’s F-12 medium before hormone treatment. After 24 h of culture, the medium was replaced with either serum-free DMEM/Ham’s F-12 medium with estradiol (10^−8^ M; Sigma-Aldrich) alone or estradiol (10^−8^ M) + dienogest (10^−6^ M; Santa Cruz Biotechnology, Dallas, TX). The ESCs treated without dienogest were used as a control (Control group), and those treated with dienogest were allocated to the Dienogest group.

Since endometriosis is an estrogen-dependent disease, the ESCs of the Control group were cultivated with estradiol at a physiological concentration (10^−8^ M). The ESCs of the Dienogest group were cultivated with estradiol at the same concentration as that used for the Control group together with dienogest at a concentration (10^−6^ M) matching that in previous studies [11–13]. After these hormonal treatments for 48 h, the ESCs were directly lysed on the culture dishes with TRIzol™ reagent (Invitrogen), immediately snap-frozen, and stored at −80 °C until further processing.

### RNA Isolation

Total RNA was purified from the cell lysates of ESCs using the RNeasy Mini Kit (Qiagen, Valencia, CA) in accord with the manufacturer’s instructions. The quantity and quality of the purified RNA were measured and assessed using a Nanodrop ND-1000 spectrophotometer (Thermo Fisher Scientific, Waltham, MA) and an Agilent Bioanalyzer (Agilent Technologies, Santa Clara, CA) before cRNA amplification and labeling.

The following processes of cRNA amplification and labeling, the sample hybridization, and the microarray data analysis were performed by a slightly modified version of the protocol described by Yokoi et al. [14].

### cRNA Amplification and Labeling

Total RNA was amplified and labeled with cyanine 3 (Cy3) using the Agilent Low Input Quick Amp Labeling Kit, one color (Agilent Technologies), following the manufacturer’s instructions. Briefly, 100 ng of total RNA was reversed-transcribed to double-stranded cDNA using a poly(dT-T7) promoter primer. The primer, template RNA, and quality-control transcripts of known concentration and quality were first denatured at 65 °C for 10 min and incubated for 2 h at 40 °C with 5× first strand buffer, 0.1 M DTT, 10 mM dNTP mix, and AffinityScript RNase Block Mix (Agilent Technologies). The AffinityScript enzyme was inactivated at 70 °C for 15 min.

The cDNA products were then used as templates for in vitro transcription to generate fluorescent cRNA. The cDNA products were mixed with a transcription master mix in the presence of T7 RNA polymerase and Cy3-labeled CTP and incubated at 40 °C for 2 h. Labeled cRNAs were purified using Qiagen’s RNeasy mini-spin columns and eluted in 30 μl of nuclease-free water. After amplification and labeling, the cRNA quantity and the incorporation of cyanine were determined using the Nanodrop ND-1000 spectrophotometer and the Agilent Bioanalyzer.

### Sample Hybridization

For each hybridization, 600 ng of Cy3-labeled cRNA was fragmented and then hybridized at 65 °C for 17 h with an Agilent SurePrint G3 Human GE v2 8×60K Microarray (Design ID: 039494). After washing, the microarrays were scanned using an Agilent DNA microarray scanner.

### Analysis of Microarray Data for the GO and Pathway Analyses

The intensity values of each scanned feature were quantified using Agilent Feature Extraction software ver. 10.7.3.1, which performs background subtractions. We used only features that were flagged as “no errors” (present flags), and we excluded features that were not positive, not significant, not uniform, not above the background, saturated, or population outliers (marginal and absent flags). Normalization was performed using Agilent GeneSpring GX ver. 11.0.2 software (per chip: normalization to the 75th percentile shift; per gene: normalization to the median of all samples). There are a total of 50,599 probes on the Agilent SurePrint G3 Human GE v2 8×60K Microarray (Design ID: 039494) without control probes. The RNA samples of the Control group were used as the total RNA reference.

Genes that were differentially expressed between the Dienogest and Control groups with a corrected *p*-value < 0.05 and absolute fold change > 1.5 were considered to be significantly differentially expressed. The differentially expressed genes were used for a gene ontology (GO) analysis within the category of biological processes by Panther software ver. 14 (Panther Labs, San Francisco, CA). The microarray data were also used for an Ingenuity® pathway analysis (IPA; Ingenuity Systems, www.ingenuity.com) to analyze canonical pathways. Fisher’s exact test was used to calculate a *p*-value to determine the significance of findings in the GO and Ingenuity® pathway analyses, and *p*-values < 0.05 were accepted as significant.

### Quantitative RT-PCR

Total RNA purified for the microarray analysis was also used for a quantitative real-time reverse-transcription polymerase chain reaction (RT-PCR) to validate the microarray data. All five RNA samples used for the microarray analysis were used for this validation experiment. One microgram of total RNA was reverse-transcribed into first-strand cDNA by using a first-strand cDNA synthesis kit, ReverTra Ace-α (Toyobo, Osaka, Japan), with random primers. The cDNA was stored at −20 °C until use.

Of the genes that were differentially expressed between the Dienogest and Control groups, we selected colony-stimulating factor 1 (CSF1), macrophage stimulating 1 (MST1), MMP-1, MMP-3, MMP-10, and tissue inhibitors of metalloproteinase-4 (TIMP-4) for the PCR reactions, which were performed in an ABI 7300 Real-Time PCR System (Applied Biosystems, Carlsbad, CA) using the KAPA SYBR® FAST qPCR kit (Nippon Genetics, Tokyo) under thermal cycling conditions in accord with the manufacturer’s instructions. The primer sequences of CSF1, MST1, MMPs, and TIMP-4 used in the present analysis were the same as those designed in previous studies [15–17]. Real-time RT-PCR for the housekeeping gene GAPDH was also performed for all of the samples to evaluate the quality of the cDNAs used. The primers used for the GAPDH quantification were designed by Wang et al. [18].

The relative quantification of gene expression for each gene was performed with the 2^−ΔΔ*C*T^ method described by Livak and Schmittgen [19]. Each *C*_T_ value was averaged for each duplicate, and then the ΔΔ*C*T value for each gene ((*C*_T_._target_ − *C*_T_._GAPDH_) _Dienogest−_ (*C*_T_._target_ − *C*_T_._GAPDH_) _Control_) was calculated. The relative expression of each gene in the Dienogest group is shown as a fold change to that in the Control group by using the equation of 2^−ΔΔ*C*T^.

### Immunohistochemistry (IHC)

Five ovarian endometriotic cysts obtained from patients treated with dienogest (Dienogest group) and five ovarian endometriotic cysts obtained from the patients not treated with dienogest (Control group) were evaluated by IHC to validate the data from the GO and Ingenuity® pathway analyses. Of the genes extracted by the GO and pathway analyses, CSF1, MST1, MMP-1, and MMP-3 were selected for the IHC analysis. Deparaffinized and rehydrated tissue sections were incubated in the PT Link pre-treatment system (Dako Agilent, Santa Clara, CA) for antigen retrieval and then processed on Autostainer Link 48 (Dako Agilent) in accord with the manufacturer’s protocol. The following primary antibodies were used: CST1 (1:100 dilution, cat.# ab52864; Abcam, Boston, MA), MST1 (1:50 dilution, cat.# HPA024036; Sigma-Aldrich), MMP-1 (1:100 dilution, cat.# 52631; Abcam), and MMP-3 (1:100 dilution, cat.# 52915; Abcam). The staining intensity was scored as follows: 0, none of the cells stained positively; 1, weak staining; 2, moderate staining; and 3, strong staining. The results were analyzed with Student’s *t*-test to compare the staining intensity for each gene between the Dienogest and Control groups, and *p*-values < 0.05 were accepted as significant.

## Results

### Identification of Genes Differentially Expressed Between the Dienogest and Control Groups

**Fig. 1:**
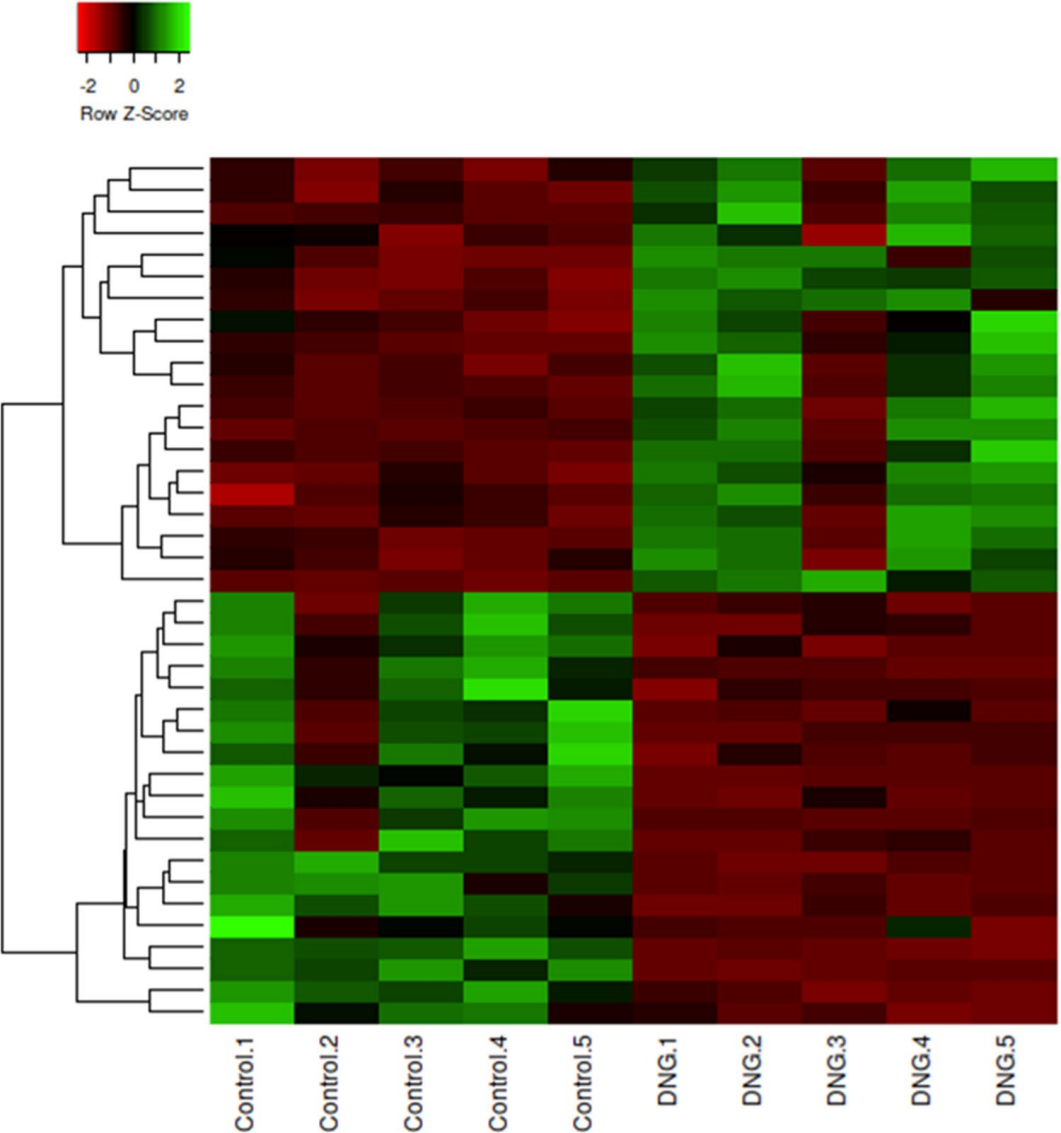
Hierarchical clustering heatmap of the top 20 upregulated and downregulated genes of the Dienogest group compared to the Control group

Under the criteria of the fold change of at least ± 1.5 with a corrected *p*-value of < 0.05, 824 genes were revealed to be differentially expressed between the Dienogest and Control groups. Of these 824 genes, 341 were upregulated and 483 were downregulated in the Dienogest group compared to the Control group levels. Tables 1 and 2 show the top 20 genes upregulated and downregulated in the Dienogest group, respectively. An overview of these genes was also created by hierarchical clustering (Fig. 1).

**Table 1:** Top 20 genes upregulated in the Dienogest group

Probe ID	Gene symbol	Fold change
A_23_P8801	CYP3A5	5.96
A_33_P3422499	Not identified	5.48
A_33_P3327673	COBL	4.84
A_19_P00318561	LOC100506516	4.7
A_24_P127691	DNAH14	4.4
A_21_P0008435	XLOC_011016	4.27
A_23_P390958	PCDHB18	4.23
A_33_P3290748	LOC648044	4.1
A_33_P3212269	MAP2K3	4.09
A_24_P7965	ESRRG	3.95
A_21_P0010916	XLOC_l2_001548	3.92
A_33_P3409518	TUBBP5	3.77
A_23_P120504	C20orf46	3.76
A_21_P0014652	LOC100507411	3.65
A_19_P00330814	HOTAIR	3.51
A_33_P3272614	PPP1R26	3.41
A_33_P3268129	Not identified	3.37
A_33_P3333156	C11orf70	3.33
A_21_P0014780	LOC100652747	3.2
A_33_P3424062	KCNF1	3.18

**Table 2:** Top 20 genes downregulated in the Dienogest group

Probe ID	Gene symbol	Fold change
A_23_P1452	NPFFR1	−6.53
A_23_P313550	SLC25A41	−5.84
A_23_P420281	PRKCB	−5.71
A_23_P329768	GREB1	−5.61
A_33_P3376273	GK	−4.77
A_33_P3409266	GAFA2	−4.16
A_32_P32905	Not identified	−3.67
A_21_P0010584	XLOC_l2_000696	−3.51
A_33_P3410659	CLEC12B	−3.49
A_24_P265088	PDZD4	−3.45
A_23_P302681	FIGNL1	−3.42
A_33_P3262376	OTUD7A	−3.31
A_24_P395814	CGB	−3.21
A_33_P3281171	LOC100130987	−3.08
A_33_P3270197	DIS3L2	−3.06
A_33_P3260342	NFASC	−2.94
A_21_P0001141	XLOC_000869	−2.91
A_21_P0010120	XLOC_013781	−2.78
A_21_P0001404	XLOC_000511	−2.74
A_21_P0010200	XLOC_014056	−2.72

### GO Analysis

The GO enrichment analysis using the Panther software was performed for the genes that were differentially expressed between the Dienogest and Control groups to reveal the biological functions of these genes. Table 3 lists the top five GO biological processes according to fold enrichment. The main biological processes identified in the GO enrichment analysis were the regulation of macrophage chemotaxis, the collagen catabolic process, and the proteoglycan biosynthetic process.

**Table 3:** Top 5 GO biological processes among the genes differentially expressed between the Diengest and Control groups

GO biological process	Gene count	Fold enrichment	Raw p-value	FDR	Involved genes
Regulation of macrophage chemotaxis	6	9.9	7.13E-05	2.29E-02	STK4, CSF1, MST1, C5AR1, MMP28, MTUS1
Collagen catabolic process	7	7.52	8.34E-05	2.53E-02	MMP3, MMP12, MMP1, MMP27, MMP25, MMP28, MMP10
Proteoglycan biosynthetic process	9	6.93	1.46E-05	8.56E-03	CSGALNACT1, FOXL1, CYTL1, B3GAT1, IGF1, HS3ST3B1, PXYLP1 HS6ST2, GAL3ST4
Regulation of cation channel activity	14	3.49	9.40E-05	2.75E-02	NEFL, CAMK2B, CACNB4, KCNG1, GPR35, GRIN2A, CACNB2, KCNAB1 JPH3, FGF13, LRRC52, CACNA1F, CACNG7, EPHB2
Calcium ion transmembrane transport	14	3.4	1.22E-04	3.33E-02	CATSPER3, CACNA1B, PKD1, PKD2L1, HTR2A, CACNB4, GRIN2A CACNB2, SLC24A1, ITPR1, CACNA1F, CACNG7, PTPRC, ATP2A3

### Ingenuity® Pathway Analysis (IPA)

**Fig. 2:**
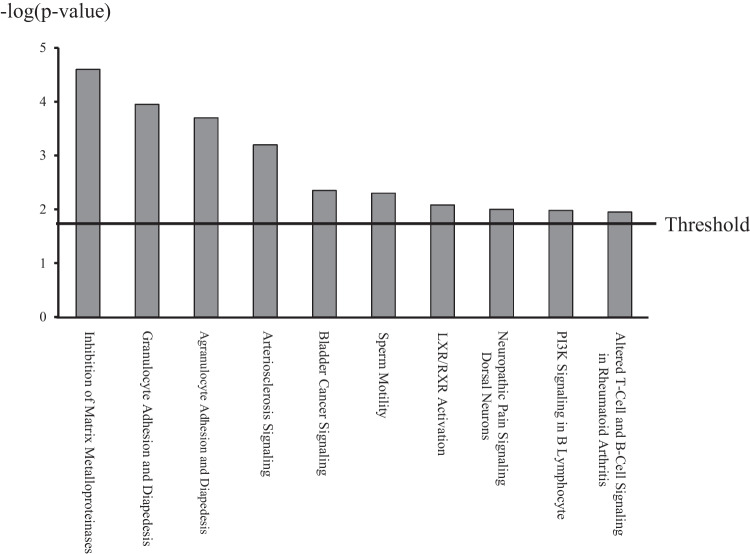
Top 10 canonical pathways most significantly differentially expressed in the Dienogest group compared to the Control group. The *transverse line* indicates the threshold of the distinct pathways in the Dienogest group under the condition of p<0.05

The IPA comparing the Dienogest and Control groups identified 20 significant canonical pathways based on 647 differentially expressed genes, which were extracted under the same conditions. Figure 2 depicts the top 10 pathways of these 20 canonical pathways. The most frequently associated genes in the 20 canonical pathways were MMPs (MMP-1, -3, -10, -12, -25, and -27). Table 4 shows the eight canonical pathways with which these MMPs were closely associated.

**Table 4:** Eight pathways with which MMPs are associated among the 20 canonical pathways. ↑: upregulated in the Dienogest group **↓**: downregulated in the Dienogest group. The bolded genes are MMPs associated with the 8 pathways

Pathway	Number of associated genes	Associated genes
Inhibition of matrix metalloproteases	7	**MMP-1↓ MMP-3↓ MMP-10↓ MMP-12↑**
		**MMP-25↓ MMP-27↓ TIMP-4↑**
Granulocyte adhesion and diapedesis	13	C5AR1↓ CCL7↑ CCL11↑ CCL25↑ CLDN3↓
		CXCL14↑ **MMP-1↓ MMP-3↓ MMP-10↓**
		**MMP-12↑ MMP-25↓ MMP-27↓** VCAM1↑
Atherosclerosis signaling	11	APOA1↑ APOB↓ CCL11↑ CSF1↓ IL6↑ **MMP-1↓**
		**MMP3↓** PLA2G2A↑ PLA2G4E↓ PON1↑ VCAM1↑
Bladder cancer signaling	7	FGF13↑ **MMP-1↓ MMP-3↓ MMP-10↓**
		**MMP-12↑ MMP-25↓ MMP-27↓**
HIFα signaling	6	**MMP-1↓ MMP-3↓ MMP-10↓**
		**MMP-12↑ MMP-25↓ MMP-27↓**
Leukocyte extravasation signaling	9	CLDN3↓ CXCL14↑ **MMP-1↓ MMP-3↓ MMP-10 ↓**
		**MMP-12↑ MMP-25↓ MMP-27↓** VCAM1↑
Role of macrophages, fibroblast and endothelial cells in rheumatoid arthritis	12	C5AR1↓ CAMK2B↓ CSF1↓ GNAO1↓ IL6↑ **MMP-1↓**
		**MMP-3↓** PLCE1↑ PLCL2↓ SOST↓ TLR3↑ VCAM1↑
Hepatic fibrosis /hepatic stellate cell activation	8	CSF1↓ HGF↓ IGF1↑ IL6↑ IL4R↓ **MMP-1↓**
		SERPINE1↑ VCAM1↑

### Quantitative RT-PCR to Validate the Microarray Data

**Fig. 3:**
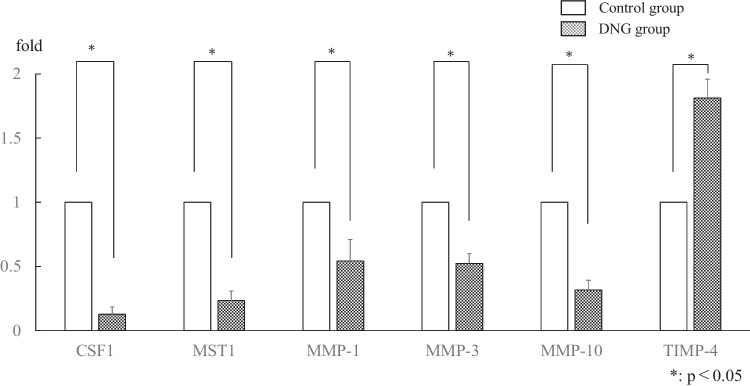
Validation of the microarray data and the pathway analysis by real-time RT-PCR by using all five RNA samples used for the microarray analysis. The expressions of CSF1, MSP1, MMP-1, MMP-3, and MMP-10 were significantly decreased whereas that of TIMP-4 was significantly increased in the Dienogest group compared to the Control group levels

Of the genes that were revealed to be differentially expressed between the Dienogest and Control groups, we selected CSF1, MST1, MMP-1, -3, and -10, and TIMP-4 for validation of the microarray data. The data illustrated in Fig. 3 demonstrate that CSF1, MSP1, MMP-1, -3, and -10 were significantly decreased in the Dienogest group compared to the levels in the Control group, whereas TIMP-4 was significantly increased.

### IHC to Validate the Data of the GO and Pathway Analyses

**Fig. 4:**
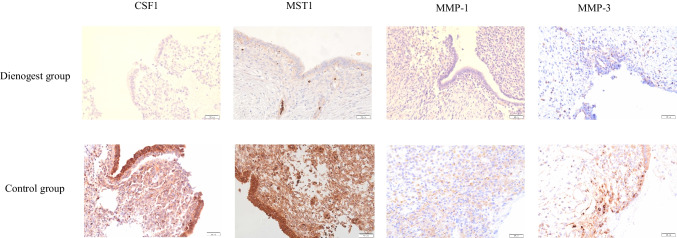
Immunohistochemical staining of CSF1, MST1, MMP-1, and MMP-3. The levels of all of these proteins were decreased in the Dienogest group compared to those in the Control group

The comparison of the expression levels of CSF1, MST1, MMP-1, and MMP-3 between the Dienogest and Control groups revealed significant differences (*p* < 0.05), with all of these proteins being expressed at lower levels in the Dienogest group compared to the Control group (Fig. 4, Table 5).

**Table 5:**
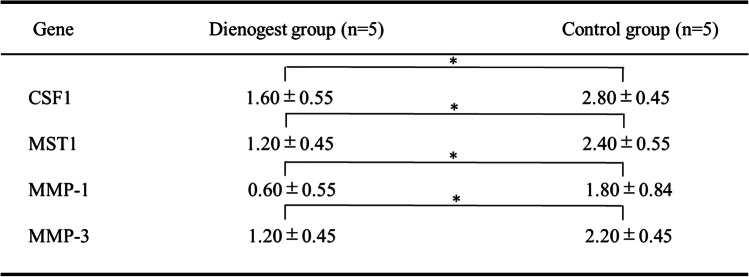
Expression levels of CSF1, MST1, MMP-1, and MMP-3. All of these proteins were expressed at significantly lower levels in the Dienogest group than in the Control group.(*p < 0.05)

## Discussion

Although dienogest has been used as the main hormonal therapy for endometriosis, its precise anti-endometriotic mechanism has remained unclear. Various investigations have shown several anti-endometriotic effects of dienogest but, to the best of our knowledge, no studies have analyzed differences in genome-wide gene expression profiles between untreated endometriotic cells and endometriotic cells treated with dienogest. In the present study, we comprehensively investigated the direct effects of dienogest on ovarian ESCs. Our findings revealed that the MMPs were the genes in the 20 canonical pathways that were most closely associated with dienogest treatment.

A set of MMPs controls physiological functions of the female reproductive tract such as ovulation, menstruation, and embryo implantation. In particular, endometrial repair, regeneration, and breakdown through the menstrual cycle are regulated by a delicate balance of MMPs and their inhibitors, TIMPs [20]. Aberrated expressions of MMPs and TIMPs in endometriotic cells or tissues have been shown as follows: Specific MMPs have been shown to be abnormally expressed in endometriotic lesions compared to the levels in eutopic endometrium. Several research groups have also observed that endometriotic cells have significantly higher expressions of MMP-1, -2, -3, -7, -9, or -10 compared to eutopic endometrial cells in in vitro or in vivo experiments, or in patients’ tissues [21–27]. Of the MMP genes differentially expressed between the present study’s Dienogest and Control groups, the expressions of MMP-1, -3, -10, -25, and -27 in ESCs were downregulated by the addition of dienogest. Earlier studies noted that MMP-1, -3, and -10 were inhibited by progesterone or progestin in endometrial explants or endometriotic cells [28–32]. Bruner et al. [33] reported that the establishment of ectopic human endometrium in nude mice was inhibited by the suppression of MMPs caused by progesterone treatment. The finding of downregulations of these MMPs by dienogest is thus likely to be reliable, and we therefore believe that MMPs play central roles in dienogest’s anti-endometriotic effects.

In general, the expression of MMP genes is induced by growth factors, hormones, and inflammatory cytokines through the MAPK pathway or NF-kB pathway [34]. The main source of these inflammatory cytokines, such as TNFα and IL-1, is thought to be macrophages [35]. Although the direct effect of dienogest on macrophages to counter endometriosis is still unknown, the results of the GO analysis in the present study suggest that dienogest exerts a suppressive effect on macrophage chemotaxis in ovarian ESCs.

CSF1, which is involved in macrophage chemotaxis, one of the enriched biological processes in the present GO analysis, has been shown to be upregulated in peritoneal endometriotic tissues compared to the level in eutopic endometrium [36]. It was also revealed to be downregulated in ovarian ESCs by dienogest in the present study. A similar finding was also reported in the mammary gland; specifically, progesterone administration together with estradiol resulted in a reduced expression of CSF1 compared to that upon estradiol administration alone [37]. As CSF1 promotes the release of proinflammatory cytokines from macrophages, and treatment with CSF1 siRNA in MCF-7 cells (a human breast cancer cell line) reduced the expression of MMPs in these cells [38], an inhibition of CSF1 release from ovarian ESCs by treatment with dienogest may suppress the MMP expression in ovarian endometriotic tissues.

CSF1R (receptor of CSF1) exerts tyrosine kinase activity and transduces its signal activated by CSF1 binding to downstream components through the MAPK pathway or PI3K-Akt pathway [39–43]. Our present findings demonstrated the suppressive effect of dienogest on the CSF1 expression in ESCs, suggesting that dienogest may directly inhibit MMP expression in these cells by suppressing CSF1.

MST1 is another gene involved in macrophage chemotaxis as identified in the present GO analysis. Matsuzaki et al. observed that MST1 and its receptor were upregulated in deep endometriotic tissues [44], and Xu et al. described elevated MST1R (receptor of MST1) expression in endometriotic tissues [45]. As MST1R also exerts tyrosine kinase activity, as does CSF1R [46], MST1 is likely to regulate MMP expression through the MAPK pathway or PI3K-Akt pathway. Indeed, MSP1 was shown to upregulate MMP-1 expression in skin fibroblasts [47]. Therefore, the suppressive effect of dienogest on MST1 expression may result in the direct suppression of MMP expression in ESCs.

TIMPs have also been revealed to be abnormally expressed in endometriotic tissues compared to normal eutopic endometrium. The expressions of TIMP-1, -2, and -3 were reported to be significantly lower in endometriotic tissues than normal eutopic endometrium [48, 49], and an experimental study showed that the addition of TIMP protein to the peritoneal cavity of nude mice prevented the establishment of endometriosis [33]. However, there are few studies describing a change in the expression of TIMP-1, -2, or -3 by an addition of progesterone, and our present findings did not provide any information about the changes in TIMP expressions by the addition of dienogest, with the exception of TIMP-4. The results of our present analyses showed that dienogest increased the expression of TIMP-4 in ESCs. Although there has been no report about the relationship between endometriosis and TIMP-4, the increase in TIMP-4 expression by treatment with dienogest may be related to the anti-endometriotic effect of dienogest.

This study has some limitations. Pawitan et al. described the relationship between the false discovery rate (FDR) and the sample size for microarray studies [50]. Since a microarray analysis with a small sample size as in the present study (*n* = 5) is susceptible to a high FDR, more falsely differentially expressed genes might have been extracted compared to the result that would be obtained with a large sample size. The present IHC also had a small sample size. A replication of the present IHC analyses using a greater number of samples would be worthwhile.

Endometriotic tissues consist of various types of cells such as ectopic endometrial epithelial, stromal, and immune cells. Inflammatory cytokines, which are produced mainly by immune cells (especially by macrophages in endometriotic tissues) are thought to play important roles in the pathogenesis of endometriosis [35]. However, the experimental system in the present study lacked immune cells, and it was thus not possible to assess the anti-endometriotic effect of dienogest through the inflammatory cytokines in this study, which used only ESCs. It is also not yet possible to recreate the exact biological environment of endometriotic tissues in an in vitro experiment such as that in the present study.

However, our findings revealed for the first time the direct effect of dienogest on ESCs by genome-wide gene expression profiling and a pathway analysis. The results of these analyses demonstrated that MMPs were associated with the genes in the 20 canonical pathways that were most closely associated with dienogest treatment. The results of our GO analysis also suggest that the suppressive effects of dienogest on CSF1 and MST1, which are genes involved in macrophage chemotaxis, may result in the decreased expression of MMPs in ESCs.

In line with previous studies [11–13, 51] in which the concentration of dienogest ranged from 10^-8^ M to 10^-5^ M, we used dienogest at 10^-6^ M. However, because the serum concentration of dienogest under the therapeutic dose is around 10^-7^ M [52], our experimental conditions may not reflect those in a clinical context.

Our results strongly suggest that MMPs play important roles in the therapeutic action of dienogest against endometriosis. It is clear that dienogest is useful and effective for treating endometriosis, but due to its anti-ovulatory effect, it is not used for patients who want to conceive. Patients with severe endometriosis who want to conceive more than once are likely to experience disease progression during the period without dienogest. A substrate that suppresses the expression of MMPs downstream of dienogest and does not suppress ovulation is desired, because such a substrate could conceivably be used as a drug to treat patients with endometriosis who want to conceive.

Dienogest has biological actions that are similar to those of progesterone, but dienogest and progesterone are also likely to have some differences, like other progestins. These differences remain unknown, but the setup of the present experiment prevents such differences from being determined. Other studies pursuing these differences may deepen our understanding of the precise biological actions of dienogest and the pathogenesis of endometriosis itself.

## Conclusions

To investigate the biological actions of dienogest in ovarian ESCs, we compared the genome-wide gene expression profiles between Dienogest and Control groups and extracted 824 genes that are differentially expressed between these two groups. The data revealed by the GO analysis and Ingenuity® pathway analysis using the differentially expressed genes suggested that MMPs play important roles in the anti-endometriotic effect of dienogest on ovarian ESCs. Our findings also suggest that the suppression of these MMPs by dienogest may be due to the suppression of components involved in macrophage chemotaxis, such as CSF1 and MST.

## Data Availability

The datasets used and analyzed in this study are available from the corresponding author on reasonable request.

## Abbreviations

ESC: endometriotic stromal cell
GnRH: gonadotropin-releasing hormone
IPA: ingenuity pathway analysis
MMP: matrix metalloproteinase
TIMP: tissue inhibitor of metalloproteinase
CSF1: colony-stimulating factor 1
MST1: macrophage stimulating 1
